# Supplementary material for: Crosstalk between age accumulated DNA-damage and the SIRT1-AKT-GSK3ß axis in urine derived renal progenitor cells
Source: Aging (Albany NY). 2022 Sep 24;14(20):8179–204. doi: 10.18632/aging.204300 (PMC9648809; doi:10.18632/aging.204300)
Supplement: Supplementary Figures [file aging-14-204300-s001.pdf]

SUPPLEMENTARY FIGURES

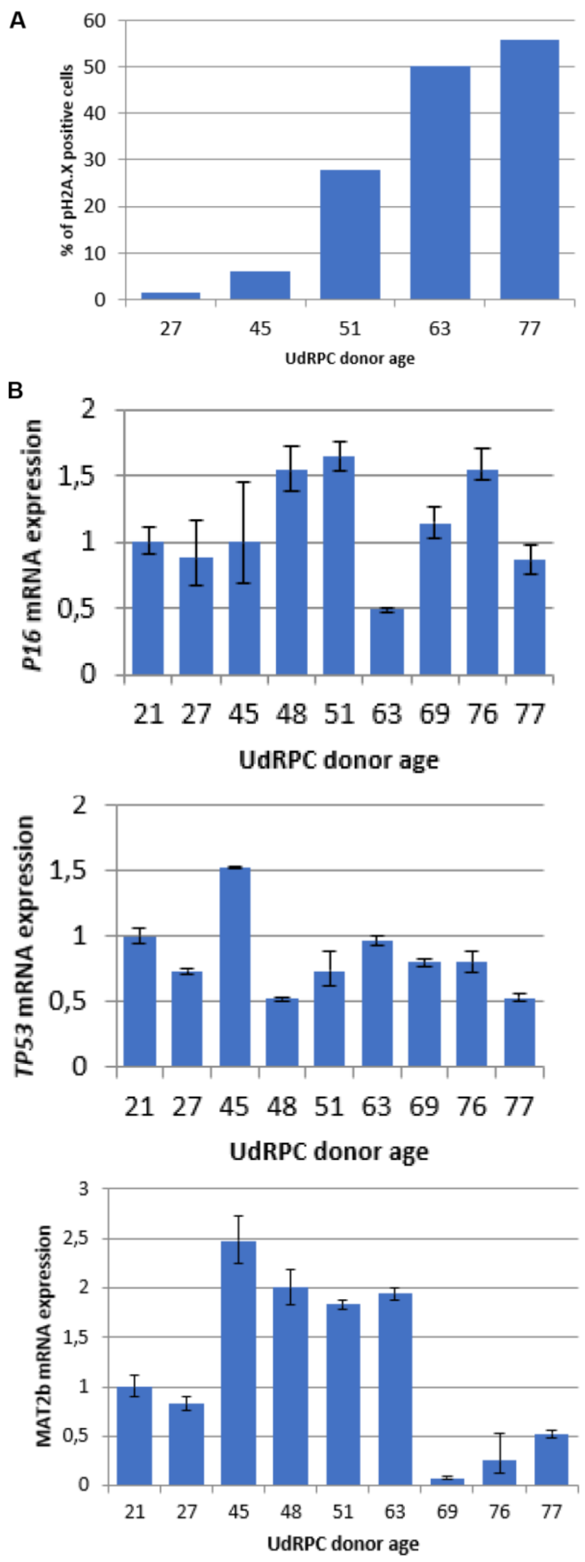

Supplementary Figure 1. UdRPCs show an increase of DNA damage with increase donor age. Percentage of pH2A.x positive cells derived from donors with different age (**A**). mRNA expression of P16, TP53 and MAT2b was determined by quantitative real time PCR (**B**).

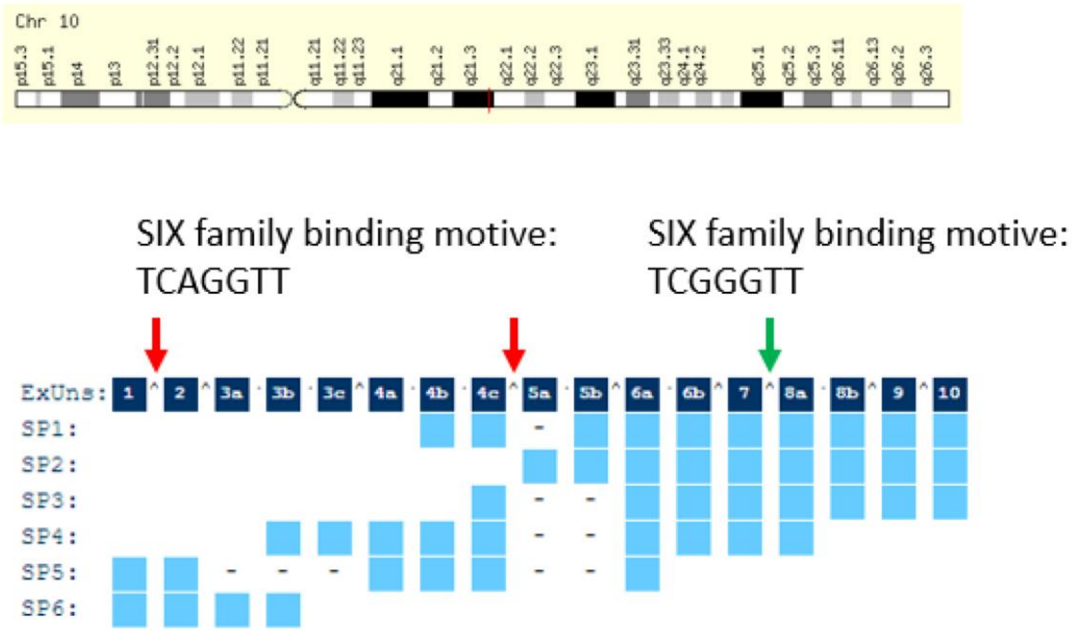

Supplementary Figure 2. SIX2/SIRT1/AKT/GSK3β network is altered in UdRPCs derived from aged donors.

A

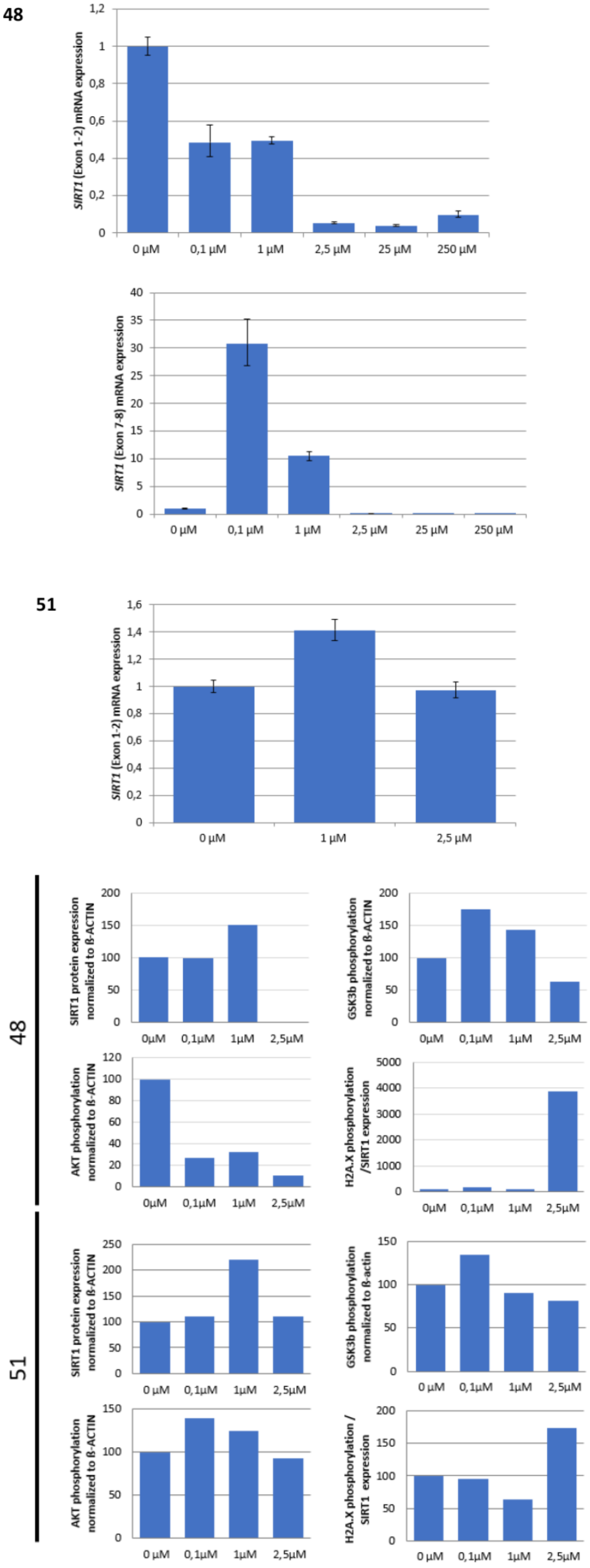
48
SIRT1 (Exon 1-2) mRNA expression
0 µM
0,1 µM
1 µM
2,5 µM
25 µM
250 µM
SIRT1 (Exon 7-8) mRNA expression
51
SIRT1 (Exon 1-2) mRNA expression
SIRT1 protein expression normalized to ß-ACTIN
GSK3b phosphorylation normalized to ß-ACTIN
AKT phosphorylation normalized to ß-ACTIN
H2A.X phosphorylation /SIRT1 expression
GSK3b phosphorylation normalized to ß-actin
H2A.X phosphorylation / SIRT1 expression
0µM
0,1µM
1µM
2,5µM

B

48

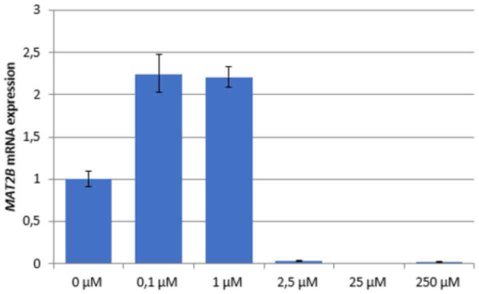

51

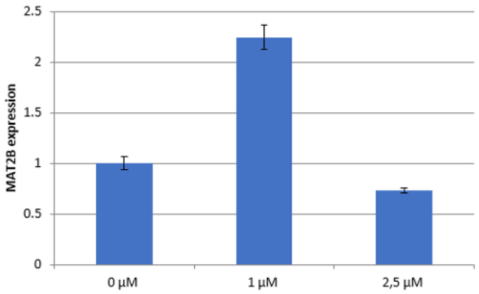

51

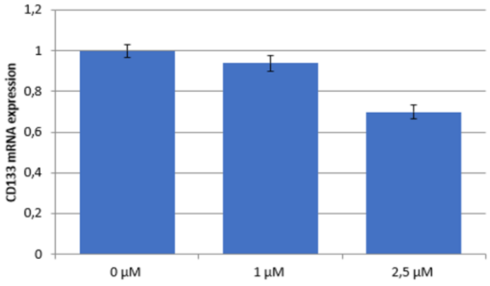

48

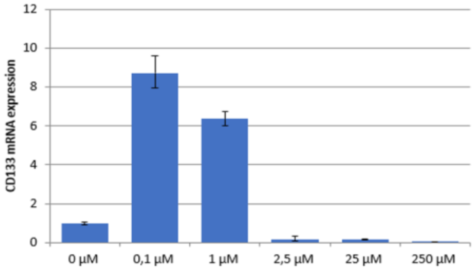

48

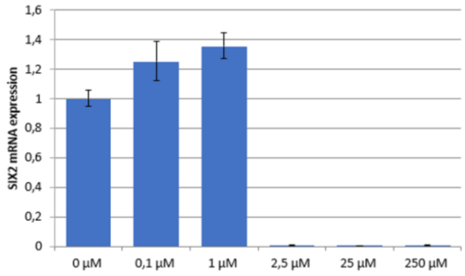

48

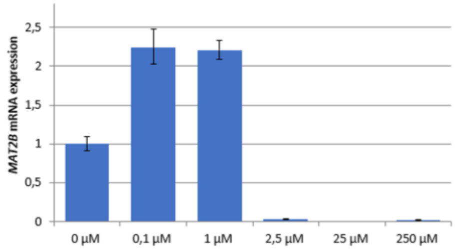

48

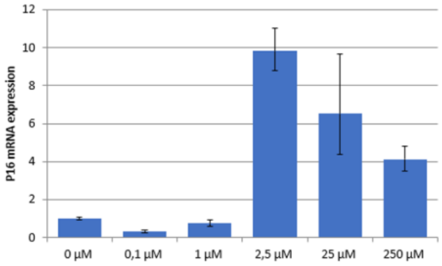

48

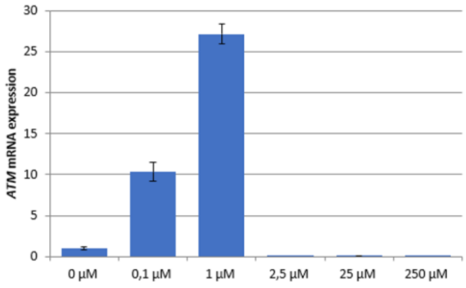

Supplementary Figure 3. The SIX2/SIRT1/AKT/GSK3ß network can be activated by resveratrol and regulates the cell fate of UdRPCs. UdRPCs derived from a 48- and 51-year-old donor were treated with different concentrations of resveratrol. Relative protein expression for SIRT1 and relative protein phosphorylation for AKT, GSK3β and pH2A.X was detected by Western blot and is given in % (**A**). mRNA expression of SIRT1, MAT2B, CD133, ATM, SIX2 and P16 was determined by quantitative real time PCR (**A**, **B**).

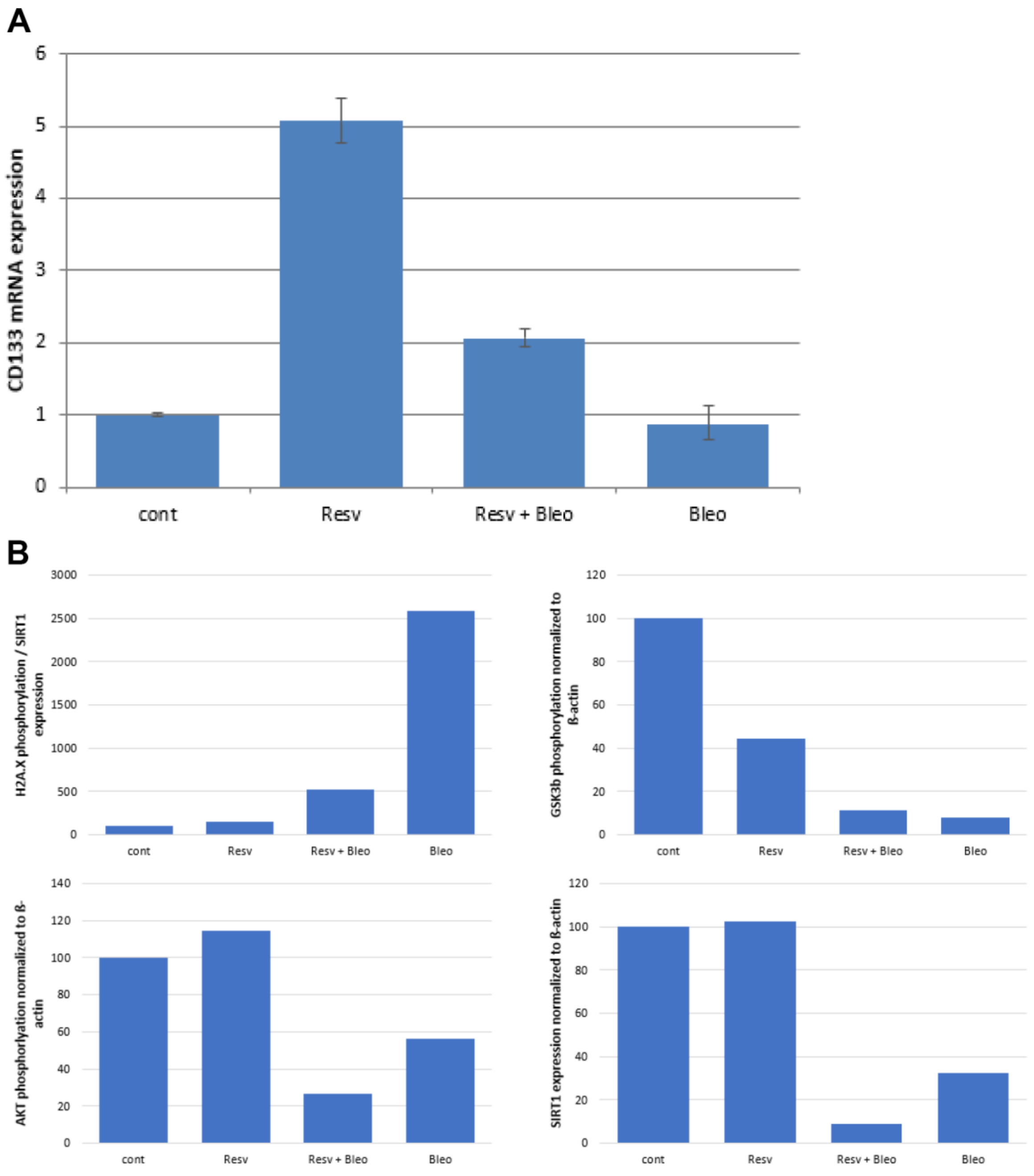

Supplementary Figure 4. DNA damage induces an aging phenotype by downregulation of SIRT1. UdRPCs derived from a 27-year-old donor were treated with resveratrol and or bleomycin. mRNA expression of CD133 was determined by quantitative real time PCR (**A**). Relative protein expression for SIRT1 and relative protein phosphorylation for AKT, GSK3β and pH2A.X was detected by Western blot and is given in % (**B**).

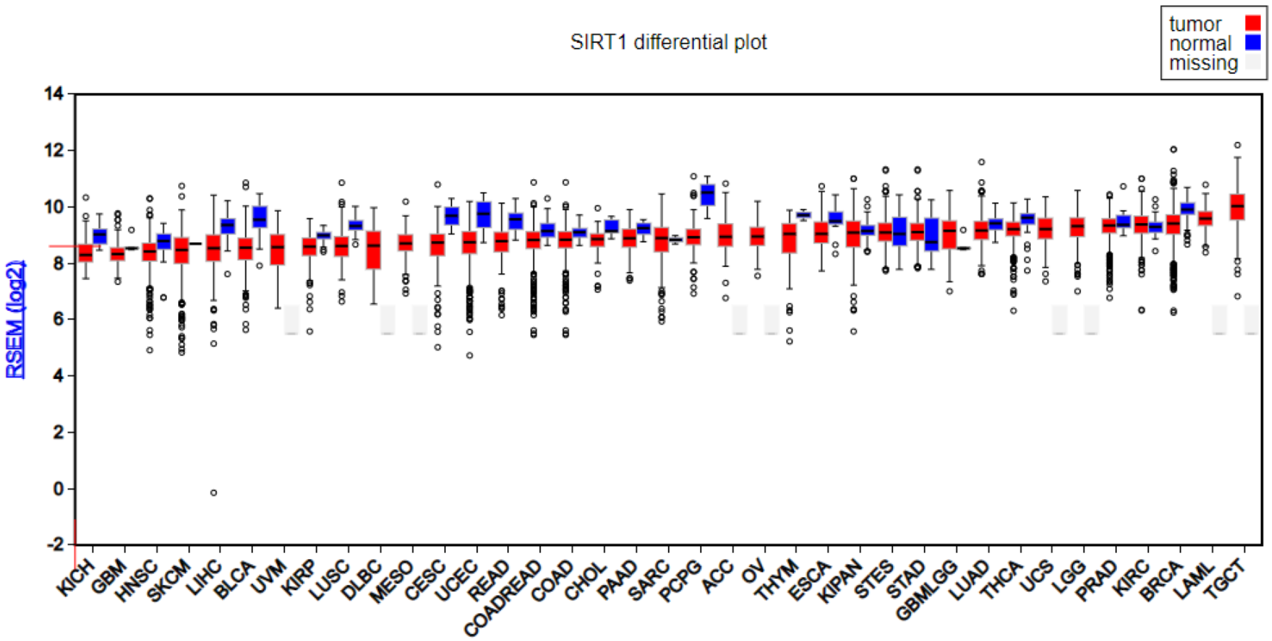

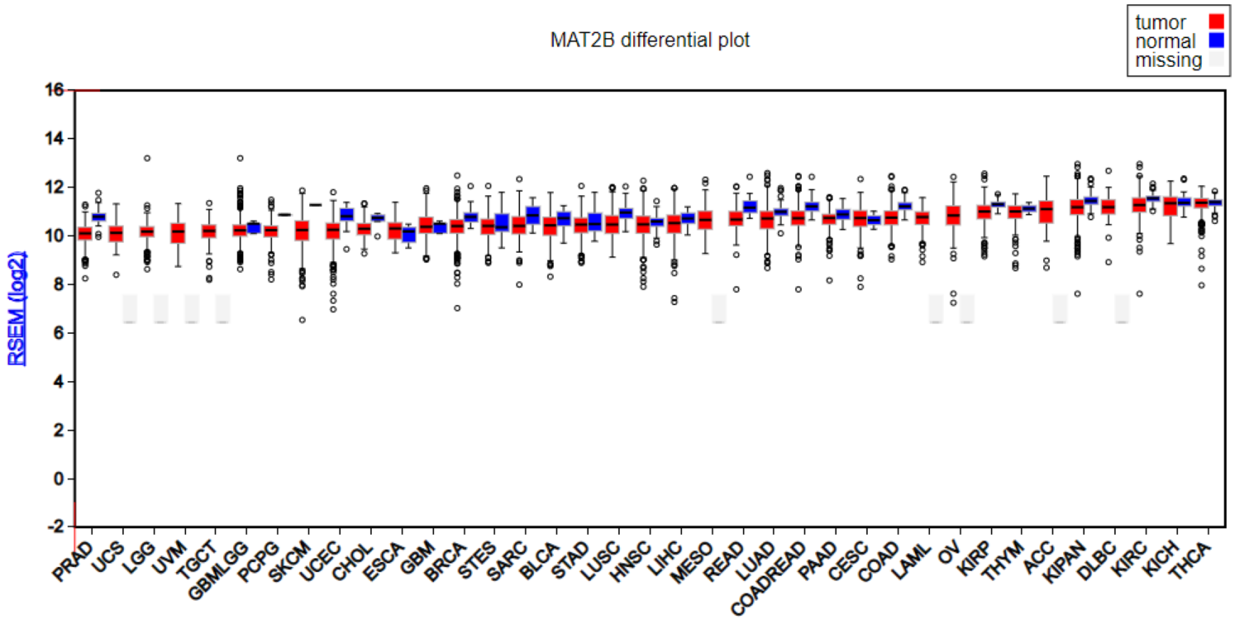

Supplementary Figure 5. TCGA SIRT1 and MAT2B expression plots.

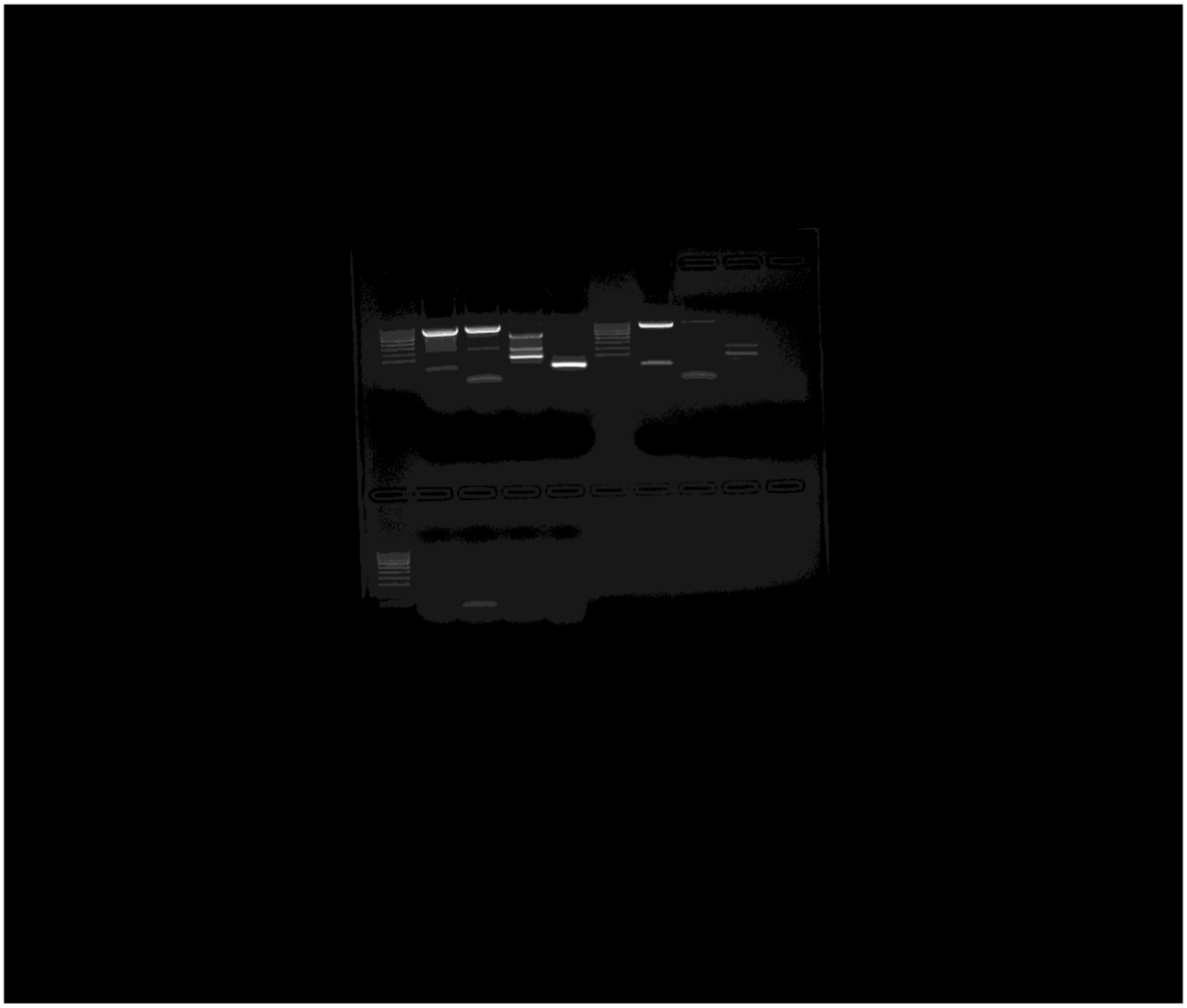

Supplementary Figure 6. Full sized gel image for immunoprecipitation PCR analysis.
