# Supplementary material for: Crosstalk between age accumulated DNA-damage and the SIRT1-AKT-GSK3ß axis in urine derived renal progenitor cells
Source: Aging (Albany NY). 2022 Sep 24;14(20):8179–204. doi: 10.18632/aging.204300 (PMC9648809; doi:10.18632/aging.204300)
Supplement: Supplementary Tables 1 and 2 [file aging-14-204300-s002.pdf]

SUPPLEMENTARY TABLES

Supplementary Table 1. RT-qPCR primers.

Primer name	Sequence	Annealing temperature (° C)	Product length (bp)
ATM s	5’-AGCTCGGATGCTTTCCTCAA-3’	60	
ATM as	5’-CTCCATCGAGAAGGTCCACG-3’		
CD133 s	5′-GACTTGCGAACTCTCTTGAATGA-3′	60	222
CD133 as	5′-GGTAGTGTTGTACTGGGCCAAT-3′		
CDKN2A s	5’-CAACGCACCGAATAGTTACG-3’	60	
CDKN2A as	5’-AGCACCACCAGCGTGTC-3’		
GDNF s	5’-GTCAAGAGAGGGTTTTCGGGT-3’	60	105
GDNF as	5’-ATCTTAAAGTCCCGTCCGGC-3’		
LMNA (Exon 9-12) s	5′-GGCTGCGGGAACAGC-3′	60	500
LMNA (Exon 9-12) as	5′-CTGGCAGGTCCC-3′		
MAT2b s	5‘-ACAGAGAGGAAGACATACCAG-3‘	60	324
MAT2b as	5‘-GTTCATTGCCAGACCAGTG-3‘		
OSR1 s	5‘-GCTAAAGCCCCAGAGACGTG-3‘	60	80
OSR1 as	5‘-TTCGGTAGTTGCAGTGGCTT-3‘		
RPL0 s	5‘-TCGACAATGGCAGCATCTAC-3‘	60	195
RPL0 as	5‘-ATCCGTCTCCACAGACAAGG-3‘		
SIRT1 (Exon 1-2) s	5‘-AGGGCGAGGAGGAGGAAGAG-3‘	60	122
SIRT1 (Exon 1-2) as	5‘-GGCTCTATCCTCCTCATCACTTTC-3‘		
SIRT1 (Exon 7-8) s	5‘-GCAGATTAGTAGGCGGCTTG-3‘	60	152
SIRT1 (Exon 7-8) as	5‘-TCTGGCATGTCCCACTATCA-3‘		
SIRT1 bseq s	5‘-GAGGGAGGAGGGTTAGAGAG-3‘	55	341
SIRT1 bseq as	5‘-CATTATCTCCTTCCCCAACC-3‘		
SIX2 s	5′-GGTATTATGTTTATGTTGTTTAT-3′	60	232
SIX2 as	5′-AACTAATAACTCTCCAAAATCT-3′		

Supplementary Table 2. Antibodies.

Antigen	Company	Dilution (IF/WB)
Mouse ß-Actin	Cell Signaling #3700	n.a./1:5000
Rabbit pAKT (Ser473)	Cell Signaling #9271	n.a./1:1000
Rabbit total AKT	Cell Signaling #9272	n.a./1:1000
Rabbit pGSK3ß (Ser9)	Cell Signaling #5558	n.a./1:1000
Rabbit total GSK3ß	Cell Signaling #5676	n.a./1:1000
Rabbit pH2A.X (Ser139)	Cell Signaling #9718S	1:200/1:1000
Mouse TP53	Merck Millipore #OP43	n.a./1:1000
Mouse SIRT1	Abcam #ab110304	1:200/1:1000
Mouse SIX2	Abnova #H00010736-1101	1:200/n.a.
Anti-mouse Alexa Fluor 488	Invitrogen #A11070	1:500
Anti-rabbit Alexa Fluor 555	Invitrogen #A21424	1:500
Anti-mouse HRP-labeled	Thermo Fisher Scientific #NA931	1:4000
Anti-rabbit HRP-labeled	Cell Signaling #7074S	1:1000
